# Dual-wavelength photoacoustic imaging of sentinel lymph nodes in patients with melanoma and breast cancer

**DOI:** 10.1016/j.pacs.2025.100747

**Published:** 2025-06-28

**Authors:** Jonas J.M. Riksen, Antonius W. Schurink, Kalloor Joseph Francis, Cornelis Verhoef, Dirk J. Grünhagen, Gijs van Soest

**Affiliations:** a Erasmus University Medical Center, Department of Cardiology, Rotterdam, the Netherlands; b Erasmus MC Cancer Institute, Department of Surgical Oncology, Rotterdam, the Netherlands; c Delft University of Technology, Faculty of Mechanical Engineering, Dept. of Precision Microsystems Engineering, Delft, the Netherlands; d Massachusetts General Hospital, Wellman Center for Photomedicine, Boston, MA, USA

**Keywords:** Photoacoustic imaging, Sentinel lymph node, Melanoma, Breast cancer, Clinical translation, Diagnostic imaging, Sentinel lymph node mapping, Oncology

## Abstract

Sentinel lymph node (SLN) biopsy is an essential procedure for accurate disease staging and treatment planning in patients with melanoma and breast cancer. Conventional preoperative imaging primarily utilizes lymphoscintigraphy with technetium-99m (Tc-99m), which presents several limitations, including radiation exposure, logistical challenges, and potential delays in surgical workflow. Photoacoustic imaging (PAI) has emerged as a promising alternative, leveraging optical contrast provided by indocyanine green (ICG). A feasibility study was conducted at Erasmus MC, University Medical Center Rotterdam, to assess the potential of dual-wavelength PAI for SLN mapping. PAI was employed to perform spectroscopic measurements in healthy volunteers, supporting the development of an optimal excitation protocol. Subsequently, in the patient phase, SLN mapping was performed using PAI with ICG, and the results were compared to the standard-of-care method utilizing Tc-99m. The excitation wavelengths of 800 nm and 860 nm were selected for ratiometric imaging to effectively visualize ICG while suppressing clutter from hemoglobin and melanin. Among the eleven evaluated sentinel nodes, seven were successfully identified using PAI. The maximum SLN detection depth achieved with PAI was 22 mm. This study illustrates the feasibility of ICG-enhanced dual-wavelength PAI for preoperative SLN mapping in patients with melanoma and breast cancer, as an alternative to lymphoscintigraphy. Analysis of false-negative detections suggests improvements to PAI and optimal patient selection. The proposed ratiometric PAI methodology, compared to multiwavelength spectroscopic imaging, enables faster imaging speeds and facilitates the transition to cheaper light sources.

## 1 Introduction

Cancer remains a leading cause of death worldwide, with estimates suggesting that about one in five people will be diagnosed with cancer in their lifetime[1]. The prognosis varies significantly by stage, with early stage localized disease associated with a highly favorable outcome, whereas advanced stages with nodal metastases carry a significantly poorer prognosis[2], [3]. This underscores the critical importance of accurate staging in guiding treatment decisions and predicting patient outcomes. For various cancers, such as melanoma and breast cancer sentinel lymph node biopsy (SLNB) is routinely performed in patients with clinically node-negative disease to detect occult nodal metastases. It plays a crucial role in accurate disease staging, prognostic stratification, and determining eligibility for adjuvant therapies (e.g immunotherapy)[3], [4]. During the procedure, the sentinel lymph node (SLN), the initial lymph node that directly receive lymphatic drainage from a primary tumor site, is surgically excised and examined through histopathological assessment for the presence of nodal metastasis.

The current localization of the SLN is performed by preoperative lymphoscintigraphy or single-photon emission computed tomography (SPECT) using a Tc-99m-nanocolloid tracer injected around the primary tumor site from where it drains towards the SLN. Intraoperative SLN localization is performed using a handheld gamma probe[5]. However, the use of Tc-99m comes with several downsides, such as radiation exposure and costs. Additionally, contrast administration poses a logistical challenge. According to Dutch guidelines, it should be injected at least 3 h, but no more than 24 h before the surgical procedure, to ensure good uptake, avoid dissociation of the nanocolloid and the radiotracer, and prevent washout of the SLN[6]. As a consequence, the patient has to arrive at the hospital well in advance in order to receive the tracer injection, with limited 'sentinel node slots' available at Nuclear Medicine department. In the case of unexpected events that lead to operating room overruns, rescheduling the procedure is challenging, limiting the number of SLNBs that can be performed daily.

Optical imaging techniques, such as near-infrared fluorescence (NIRF) using indocyanine green (ICG), could offer an alternative. ICG is a non-radioactive dye that has been widely used for SLN mapping and intraoperative surgical navigation[7], [8], [9], offering accumulation in the SLN comparable to that of radiocolloid uptake[10], [11]. However, the opacity of the skin restricts the transcutaneous detection of SLNs by ICG-NIRF to superficial nodes[12], [13]. ICG-based NIRF imaging is highly effective for intraoperative visualization of the SLN, but falls short in preoperative identification through the skin, highlighting a need for imaging methods with a deeper penetration depth.

Photoacoustic imaging (PAI) in combination with ICG could be a viable option to replace the use of Tc-99m-based preoperative imaging, while the fluorescence properties of ICG could concurrently be used for intraoperative detection. PAI has previously been utilized to detect the presence of ICG in lymphatic vessels and has demonstrated greater imaging depths compared to NIRF[14], [15]. Furthermore, SLN detection using multispectral PAI, with 6 wavelengths, was reported to achieve detection rates comparable to those of lymphoscintigraphy with Tc-99m[16]. Limiting the number of wavelengths would allow for more affordable light sources, such as diode lasers or LEDs, as the desire for wavelength flexibility comes at a great cost in terms of expense, bulk, ambient noise, and energy inefficiency in the tunable lasers required[17]. Additionally, reducing the number of wavelengths enhances the imaging frame rate, as the different wavelengths have to be applied sequentially in multispectral PAI. This concern is compounded by the low pulse repetition frequency of powerful optical parametric oscillator (OPO)- based laser systems, which affects scan time and scan stability.

This study explores the use of PAI to detect SLNs for patients with melanoma and breast cancer, aiming to use a minimum number of wavelengths to achieve an imaging approach that can potentially be translated into the routine clinical workflow of SLNB. We performed spectroscopic measurements of SLNs in healthy volunteers administered with ICG to determine the optimal set of wavelengths. With the resulting set of wavelengths, SLNs are imaged to assess the impact of administration timing. The optimized methodology is subsequently demonstrated in patients with melanoma and breast cancer and compared to conventional nuclear imaging and intraoperative NIRF imaging. By using only two wavelengths, the proposed method achieves high frame rates and real-time reconstruction, enhancing translational potential compared with multi-wavelength PAI approaches.

## 2 Methods

A feasibility study investigating PAI to image SLNs, using ICG as a contrast agent, was conducted at the Erasmus MC, University Medical Center Rotterdam. The study, which included both healthy volunteers and patients with melanoma or breast cancer, received approval from the institutional medical ethics committee (NL86093.078.24). The study is registered at the Central Committee on Research Involving Human Subjects under trial ID NL-OMON56703 and is available on the Overview of Medical Research in the Netherlands platform. All PAI in this study was carried out by the same physician (A.W.S).

### 2.1 Volunteer phase

Healthy volunteers, aged 18 years and older, with no history of lymph node surgery, who provided informed consent, were included. They subsequently underwent intradermal ICG (Verdye, Renew Pharmaceuticals Ltd, Ireland; 5 mg dissolved in 1 ml sterile water) administration, followed by massaging for approximately 5 min. The PAI measurements were performed after ICG administration at time points varying from 5 to 30 min (Supplementary Figure 1).

NIRF imaging, using the Quest Spectrum Platform (Quest Medical Imaging, Wieringerwerf, the Netherlands), was employed to identify the SLN basin by tracking the fluorescent signal of the lymphatic vessels. Once the SLN basin was identified leveraging the larger field of view of NIRF, a PAI system was employed to detect the SLN. In the first three volunteers, the identification was performed using wavelengths of 820 nm and 940 nm[18]. Following detection, spectroscopic measurements were conducted to determine the optimal wavelengths for ICG detection in the lymph nodes. Based on these spectral measurements, two wavelengths were selected that provided stronger contrast for dual-wavelength ratiometric imaging of the SLN and applied during the patient phase. Spectroscopic PAI was performed for all volunteers.

One volunteer presented for a second imaging session between 9 days after the initial experiment, to assess the persistence of the ICG signal. No new ICG was injected.

### 2.2 Patient phase

After conducting imaging in healthy volunteers, the same imaging protocol was applied to patients with melanoma or breast cancer who were scheduled for SLNB. This allowed for the validation of PAI findings against the standard care for SLN detection, conventional Tc-99m-based scintigraphy or SPECT. SPECT was used for patients with melanomas located in the head and neck region, while lymphoscintigraphy was employed for melanomas in other anatomical locations according to the standard of care. For melanomas in the head and neck, a Tc-ICG dual tracer was used as the standard of care, with the dose of ICG ranging from 0.01 to 0.04 mg[13].

PAI dual-wavelength measurements were performed following Tc-99m injection and prior to nodal imaging. Researchers were blinded to the location and number of SLNs, and the study time was limited to 30 min for logistical reasons. A clinically accepted dose of 5 mg ICG (Verdye, Renew Pharmaceuticals Ltd, Ireland), dissolved in 1 ml sterile water, was intradermally injected, followed by massaging for approximately 5 min. ICG was injected per study protocol (5 mg) or as a combined ICG-Tc-99m agent (0.01–0.04 mg ICG) in the standard of care. As we observed that detection with the combined agent was unpredictable in the first 4 patients, ICG-only was used for all following cases.

During the SLNB, decisions were made solely on the standard care, based on lymphoscintigraphy. The SLN was identified based on the Tc-99m signal, with the NIRF camera confirming the presence of ICG in the excised SLN (Supplementary Figure 1).

### 2.3 Photoacoustic imaging

The PAI system used in this investigation consisted of a Vantage 256 ultrasound system (Verasonics, Kirkland, WA, USA) and the L12–3v transducer (7.5 MHz center frequency, 93 % bandwidth, Verasonics, Kirkland, WA, USA) connected to a tunable diode-pumped Nd:YAG-OPO laser (PhotoSonus X, 100 Hz, 5 ns pulse width, Ekspla, Vilnius, Lithuania). The laser was coupled into a bifurcated fiber bundle, terminated with two slit outputs, each measuring 2.5 cm in length and 0.25 cm in width, attached to the transducer using a 3D-printed holder. The optical beams were positioned to intersect at 2 cm depth from the transducer surface. At a wavelength of 800 nm, the measured fluence on the skin was 28.1 mJ/cm2, lower than the maximum permissible exposure limits of 31.7 mJ/cm^2^. Laser safety precautions, including the use of safety goggles and a laser-safe room, were followed at all times during the measurement.

Spectroscopic measurements were performed in healthy volunteers across the wavelength range of 800–1000 nm, with a spectral resolution of 5 nm. Following pulse energy compensation (normalization per wavelength), the data was beamformed into spectroscopic imaging stacks to evaluate the PAI spectra at the SLN locations. Based on the results of the spectroscopic evaluation, we identified the combination of two optical wavelengths that maximized the contrast for ICG absorption, while the absorbance from other chromophores was similar for both.

For the dual-wavelength PAI investigation, ultrasound plane wave imaging (7 angles), as well as photoacoustic images at the optimal excitation wavelengths were acquired. Following pulse energy compensation, photoacoustic ratio images were generated after averaging both wavelengths 10 times, resulting in a real-time imaging frame rate of 5 Hz. The photoacoustic ratio images were overlaid on the ultrasound image, highlighting the presence of ICG, as well as the morphological structure.

## 3 Results

### 3.1 Volunteer phase

Eight healthy volunteers were scanned during the volunteer phase of the study. Five participants received injections in the lower extremities at least 10 cm distal to the groin to visualize the inguinal SLN, while three were injected in the upper extremities at least 10 cm from the axilla to target the axillary nodes. In all inguinal measurements, lymph nodes were clearly identifiable on ultrasound and demonstrated ICG uptake by PAI. Among the volunteers injected in the upper extremities, ICG signals were detected by PAI in the axillary lymphatic vessels in all three cases and in a lymph node in two cases. ICG was typically visible in the lymph node after 5 min of massage, with the case remeasured after 9 days still showing a detectable signal.

**Fig. 1:**
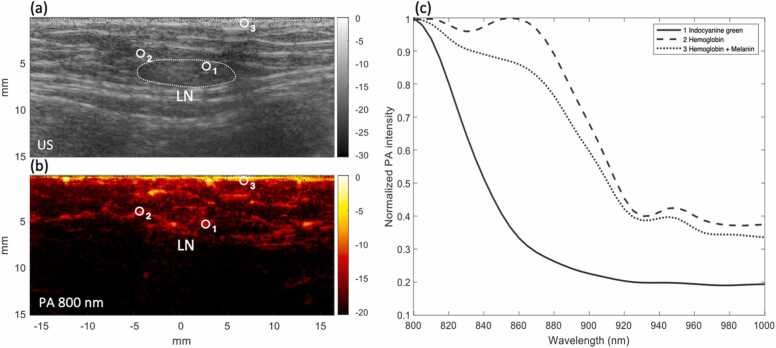
Measured photoacoustic spectra in and around a lymph node with indocyanine green contrast. The ultrasound image (a) shows a lymph node with in (b) the corresponding photoacoustic image at 800 nm. Three regions of interest are marked, and the average spectra of the pixels within each region are visualized in panel (c), which shows the normalization by max. The regions show typically measured spectra: Region 1 shows an ICG spectrum, Region 2 shows a hemoglobin spectrum, and Region 3 shows a combination of hemoglobin and melanin, as it is close to the skin surface.

Spectroscopic PAI measurements typically show spectra as depicted in Fig. 1, with dominant chromophores hemoglobin, melanin, and ICG. A specific ICG absorption peak at around 800 nm can be observed. Due to the high absorbance from lipid and water, spectral coloring begins to progressively reduce the typical photoacoustic signal at depth starting at around 900 nm. Therefore, wavelengths at 800 nm, with high ICG absorbance, and 860 nm, with low ICG absorbance but a similar hemoglobin signal, were selected for dual-wavelength ratiometric imaging. This enables the differentiation of the ICG signal from the other present chromophores.

**Fig. 2:**
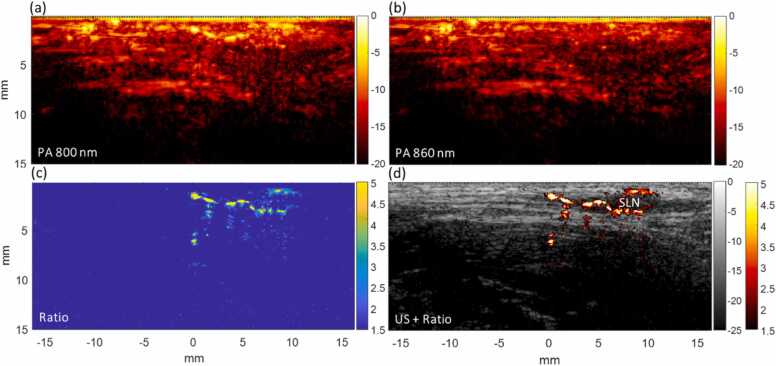
Photoacoustic signal at 800 nm (a) and 860 nm (b) of a superficial inguinal sentinel lymph node (SLN), 30 min after indocyanine green (ICG) administration. Other chromophores are present in both images, while ICG is primarily visible in the 800 nm image. An 800/860 nm ratio image (c) suppresses signal from other chromophores leaving the ICG visible with relatively high ratios. (d) Ratio image overlaid on the corresponding ultrasound image.

A typical example of this dual-wavelength imaging method, visualizing ICG in a superficial inguinal lymph node 30 min post-injection, is shown in Fig. 2. The photoacoustic images at 800 and 860 nm, in Fig. 2(a) and (b), show a large amount of clutter originating from hemoglobin and melanin. While both photoacoustic images show vascular structures, the ICG signal is present exclusively in the 800 nm image. By applying a ratio method of 800/860 nm, the signal from other chromophores is suppressed to the background, leaving the ICG signal clearly visible with high ratio values (Fig. 2(c)). This can be overlaid on the corresponding ultrasound image to show the structural information of the lymph node, as well as the ICG signal indicating it is an SLN (Fig. 2(d)).

Supplementary Figure 2, shows an inguinal SLN prior to injection where no relatively high ratio structures are imaged, but 10 min post-injection, the SLN becomes visible as ICG has accumulated.

### 3.2 Patient phase

Ten patients were included, each with an indication of SLNB. Nine patients were diagnosed with melanoma and one with breast cancer. The mean age of the group was 61 years, ranging between 37 and 77. The average BMI was 27, with a minimum of 20 and a maximum of 35. In one patient, SLNB was not performed due to the SLN location in the parotitis, and in another patient, the SLN could not be identified intraoperatively. An overview of the patient characteristics along with tumor classification and SLN status is provided in Table 1.

**Table 1:** Characteristics of enrolled patients.

No.	Age	Sex	BMI	Tumor	Tumor (T) classification	SLN status
1	63	M	24	Melanoma	T2a	-
2	60	F	25	Melanoma	T2a	Not performed
3	71	F	26	Breast cancer	T1b	Not found
4	67	F	34	Melanoma	T2a	-
5	37	M	20	Melanoma	T3a	-
6	66	M	26	Melanoma	T3a	-
7	59	F	24	Melanoma	T2a	-
8	41	F	28	Melanoma	T3a	-
9	68	F	35	Melanoma	T3b	+
10	77	M	30	Melanoma	T3b	+

M: male; F: female; BMI: body mass index; SLN: sentinel lymph node

Eleven potential lymph node basins, confirmed by nuclear imaging, were scanned and seven SLNs were identified with PAI. All detected SLNs with PAI were true positives, with no false positives recorded. In three lymph node basins where nuclear imaging confirmed the presence of SLNs, the SLN could not be detected by PAI. The observed maximum depth of approximately 22 mm was recorded during the measurements, representing the deepest ICG detection depth in our patient population. An overview of the study findings is shown in Table 2. In some cases, the lymphatic vessels were not clearly visible on NIRF imaging from the injection site, which confounded identification of the appropriate SLN basin. Combined with the limited scanning time, and the potential for multiple lymph node basins, this resulted in not all relevant SLN basins being scanned with PAI.

**Table 2:** Overview of the study findings, indicating the primary tumor location, the administered contrast agent, and the number of sentinel lymph nodes identified on nuclear imaging.

No.	Primary tumor location	Contrast agent	No. of SLNs	PAI found / SLN+ locations scanned	PAI found / SLN- locations scanned	Scanned SLN+ location	SLN depth (mm)
1	Head and neck	ICG-Tc	3	0/0	0/1		-
2	Head and neck	ICG-Tc	2	1/1	0/3	Submandibular	20*
3	Breast	Tc, ICG	0	0/0	0/3		-
4	Trunk	ICG-Tc	3	0/1	0/1	Axillary	25**
5	Trunk	Tc, ICG	1	1/1	0/1	Axillary	9*
6	Head and neck	ICG-Tc, ICG	3	1/1	0/1	Occipital	5*
7	Trunk	ICG-Tc, ICG	1	0/1	0/1	Axillary	40**
8	Upper extremity	Tc, ICG	2	0/2	-	Axilla, cubital	***
9	Lower extremity	Tc, ICG	2	2/2	-	Inguinal (2)	15*, 20*
10	Trunk	Tc, ICG	3	2/2	0/1	Axillary (2)	15*, 22*

ICG-Tc: indocyanine green technetium dual tracer; Tc: technetium; ICG: indocyanine green (5 mg); SLN: sentinel lymph node; PAI: photoacoustic imaging; *Measured using PAI; **Measurement using SPECT; ***Only scintigraphy, no depth possible.

**Fig. 3:**
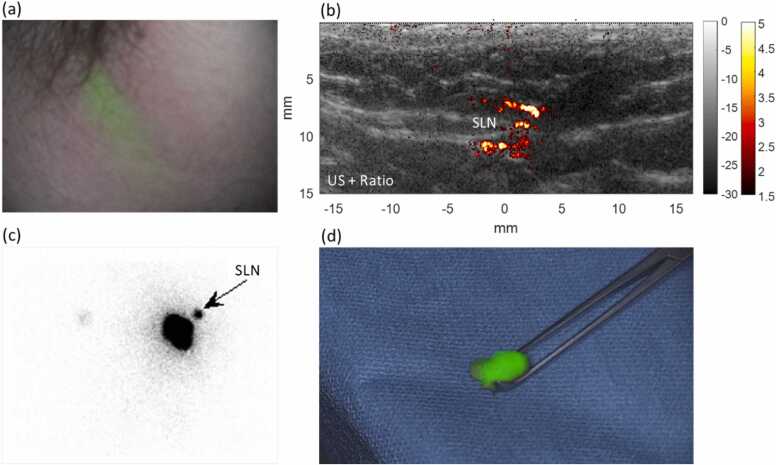
Imaging results from patient No. 5. (a) shows near-infrared fluorescence (NIRF) imaging of a lymphatic vessel indicating a sentinel lymph node (SLN) is located in the axillary basin. (b) photoacoustic ratio image overlaid on ultrasound, showing the ability to locate the SLN at a depth of approximately 10 mm, which the NIRF camera could not. The findings were confirmed with scintigraphy (c) and with intraoperative NIRF (d), confirming the PAI findings.

**Fig. 4:**
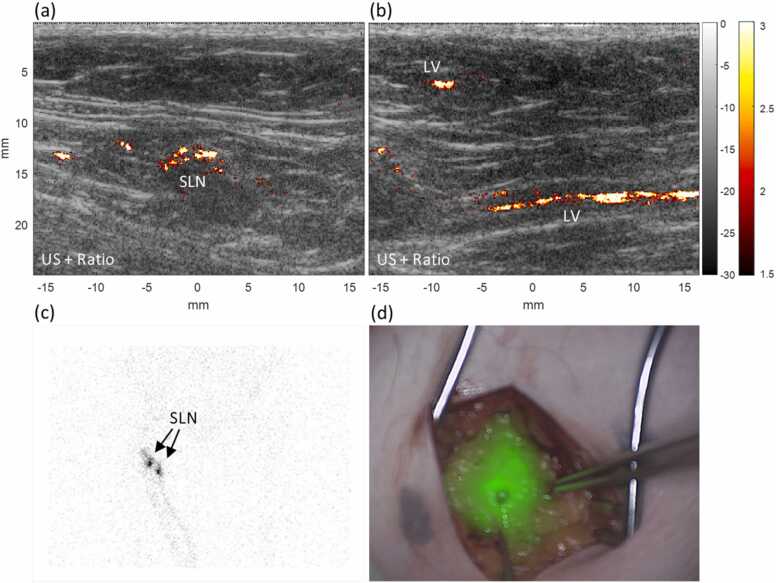
Photoacoustic imaging (PAI) results from an inguinal sentinel lymph node (SLN) and lymphatic vessel (LV) leading towards it from a patient with melanoma (patient No. 9). Ultrasound with overlaid photoacoustic ratio images of an inguinal SLN (a) and axial and parallel cross-sections of lymphatic vessels (b). Preoperative scintigraphy (c) and intraoperative NIRF imaging (d) confirmed the PAI findings.

None of the SLNs could be found by NIRF, presumably because the SLNs were located too deep. NIRF did successfully detect the superficial lymphatic vessels, allowing guidance to SLN basins. A typical example is shown in Fig. 3 (patient No. 5), where a lymphatic vessel is visualized with the NIRF camera leading to the axillary basin, but the SLN location itself could not be visualized. The PAI system could image deeper and successfully follow the lymphatic vessels leading to the exact SLN location. Another example is shown in Fig. 4 (patient No. 9) in an inguinal SLN, where high ratios are observed in an extended area, distinguishing it from lymphatic vessels, which show linear structures when parallel or dotted appearances in cross-sections relative to the transducer.

## 4 Discussion

In this study, we demonstrated that dual-wavelength PAI, using ICG as a contrast agent, can successfully identify SLNs in patients. The use of ICG as a contrast agent enables visualization of the SLN within 5 min of injection. This provides a significant advantage over conventional Tc-99m-based nuclear imaging, which requires Tc-99m injection approximately five hours before surgery to allow for adequate uptake and imaging. As result, PAI-based SLN mapping may facilitate SLNB planning, as SLN mapping can be performed immediately prior and during the surgery. In the patients included in this study, the proposed ratiometric PAI demonstrated true positive detections of SLNs, with no instances of false positives. PAI proved superior to NIRF, as none of the SLNs detected by PAI were identified by NIRF, confirming the greater imaging depth. The ability to visualize inguinal SLNs, combined with the potential for intraoperative ICG fluorescence mapping, positions PAI as a promising technique for clinical adoption.

Our findings suggest that the use of two carefully selected wavelengths, 800 and 860 nm, provided adequate information to distinguish ICG in lymph nodes. The demonstrated 800/860 nm ratio images provided real-time imaging and easy interpretation, as signals from other chromophores, like hemoglobin and melanin, are effectively suppressed, leaving only the ICG signal visible in the image (see Fig. 2). *In vivo* spectroscopic imaging of SLNs has been performed before with the Acuity Echo system (iThera Medical GmbH, Munich, Germany), showing detection frequencies equivalent to conventional scintigraphy[16]. In contrast, the PAI system used in this study employs a linear array transducer with a higher center frequency. While this ultrasound configuration results in high-quality echo images that are easy to interpret for clinical users, the higher frequency and absence of curvature in the array may limit the sensitivity to PA targets at greater depth. The imaging methodology we propose here, however, achieves successful SLN imaging with two wavelengths, enabling faster imaging speeds needed for clinical imaging, as laser repetition rates are often a limiting factor. In addition, it facilitates the transition to cheaper light sources, replacing the commonly used, bulky, and expensive tunable lasers. Efficient pulsed laser diodes are commercially available at many wavelengths in the near-infrared.

A limitation of this study was that not all SLN basins could be scanned, primarily due to limited imaging time and the complexity of certain cases. For instance, in cases involving melanomas located in the midline or located on the head or neck, which could drain to multiple lymph node basins, the available time frame was too short to cover all potential SLN basins. For PAI to be suitable for comprehensive SLN detection in such complex cases, with comparable efficacy as nuclear imaging, fast scanning of large volumes is required, this could potentially be supported by NIRF imaging. Patient characteristics and clinical presentation may further help to decide whether PAI is a suitable technique for SLN localization; inherent properties of PAI were not observed to be a limiting factor. For simpler cases, where SLN basins are more predictable, such as melanomas located on the extremities or patients with breast cancer, PAI showed promising results. Among all SLNBs performed for melanoma, approximately 25–30 % are located in the lower extremities and 15–20 % in the upper extremities[19]. The addition of NIRF imaging was able to provide some assistance in localizing the correct SLN basins but also added to the time burden.

Furthermore, we found three false-negative detections by PAI of SLNs located in basins that were confirmed by nuclear imaging, suggesting limitations of using PAI to map SLNs. Several factors may have contributed to this discrepancy, including the tracer used in the imaging process. In one case (No. 4), only the dual tracer (ICG-TC, containing 0.01–0.04 mg ICG) was used instead of 5 mg of ICG, potentially affecting PAI visualization, although a NIRF signal limiting to the injection site could be observed. In another case (No. 7), the depth constraint of PAI was evident, as SLNs located deeper than 40 mm could not be visualized due to optical attenuation and limitations of ultrasound receive sensitivity. These limitations are device-specific and could likely be improved with an imaging system optimized for PAI, and adequate dosing. Despite following similar protocols to those used in NIRF studies[13], [20], in a final case (No. 8) the ICG uptake was not detectable by NIRF and PAI during the measurement window. Other cases also showed a slow uptake of ICG to the SLN, although the SLNs were detectable 20 min after injection. These variations underscore the need for further exploration of ICG dosing, imaging protocols, and device optimization to improve the consistency and effectiveness of PAI in SLN mapping.

ICG spectroscopy is prone to variability, depending on concentration, solvent, binding proteins, and other environmental factors[21]. A dual-wavelength approach, therefore, may not capture the spectral variability, making it potentially less robust than a more comprehensive, time-consuming spectroscopic imaging approach. On the other hand, multi-wavelength spectral unmixing approaches can be more susceptible to the effects of spectral coloring than ratiometric imaging with a pair of well-chosen wavelengths[22]. In the data presented here, of SLNs and lymphatic vessels of the scanned volunteers, the ICG spectral absorption peak was found to be consistent at 800 nm, while a variable maximum absorption wavelength was observed at the injection site, which is shown in Supplementary Figure 3.

The work demonstrates measuring ICG specifically for SLN mapping in patients with melanoma and breast cancer. However, the ratio approach with 800/860 nm could also be applied in other areas where *in vivo* ICG visualizations are clinically relevant, such as SLN mapping in other cancers, tumor tissue imaging, anatomic identification, and target-specific probes[23]. The relatively simple methodology effectively moves clutter from non-relevant chromophores, like hemoglobin and melanin, to the background, enabling the visualization of solely the ICG. Overall, the imaging becomes easier for clinicians to interpret, and using just two wavelengths enables the high imaging speeds needed in a clinical setting, along with eliminating the need for tunable lasers.

## 5 Conclusion

This work demonstrated the feasibility of ICG-mediated dual-wavelength PAI for preoperative SLN mapping in patients with melanoma and breast cancer. Spectroscopic evaluation of SLNs in volunteers revealed that the two optimal wavelengths in order to distinguish ICG from other present chromophores are 800 and 860 nm. Using real-time ratiometric imaging of these two wavelengths enabled visualization of ICG in lymph nodes, confirming it as a SLN. This was demonstrated in patients undergoing SLNB, confirming the PAI findings with nuclear imaging and intraoperative NIRF imaging. A reliable ICG-based PAI signal was observed from SLNs within 20 min after injection in the majority of subjects included in this study, persisting for several days. PAI resulted in true positives without the presence of false positives. Inguinal lymph nodes with ICG were effectively identified in all volunteers and patients, suggesting the effectiveness of dual-wavelength PAI for this anatomical region.

## CRediT authorship contribution statement

**Gijs van Soest:** Writing – review & editing, Supervision, Methodology, Conceptualization. **Schurink Antonius:** Writing – review & editing, Writing – original draft, Methodology, Investigation, Formal analysis, Conceptualization. **Kalloor Joseph Francis:** Writing – review & editing, Methodology, Conceptualization. **Cornelis Verhoef:** Writing – review & editing, Supervision, Methodology, Conceptualization. **Grünhagen Dirk:** Writing – review & editing, Supervision, Methodology, Conceptualization. **Riksen Jonas:** Writing – review & editing, Writing – original draft, Visualization, Methodology, Investigation, Formal analysis, Conceptualization.

## Declaration of Competing Interest

The authors declare the following financial interests/personal relationships which may be considered as potential competing interests: Gijs van Soest is a cofounder of, and has equity in, Kaminari Medical BV, who were not involved in the submitted work. In the past three years, he was the PI on research projects, administered by Erasmus MC, that received research support from FUJIFILM VisualSonics, Shenzhen Vivolight, Boston Scientific (outside the submitted work), Waters and Mindray (related to the submitted work). Ton van der Steen is a strategic advisor of, and has a financial interest in, Kaminari Medical BV. The other authors have no conflicts of interest to declare

## Data Availability

The authors do not have permission to share data.
