# Supplementary material for: Dual-wavelength photoacoustic imaging of sentinel lymph nodes in patients with melanoma and breast cancer
Source: Photoacoustics. 2025 Jun 28;45:100747. doi: 10.1016/j.pacs.2025.100747 (PMC12269618; doi:10.1016/j.pacs.2025.100747)
Supplement: Supplementary file 1 — Supplementary material [file mmc1.docx]

# Supplementary figures


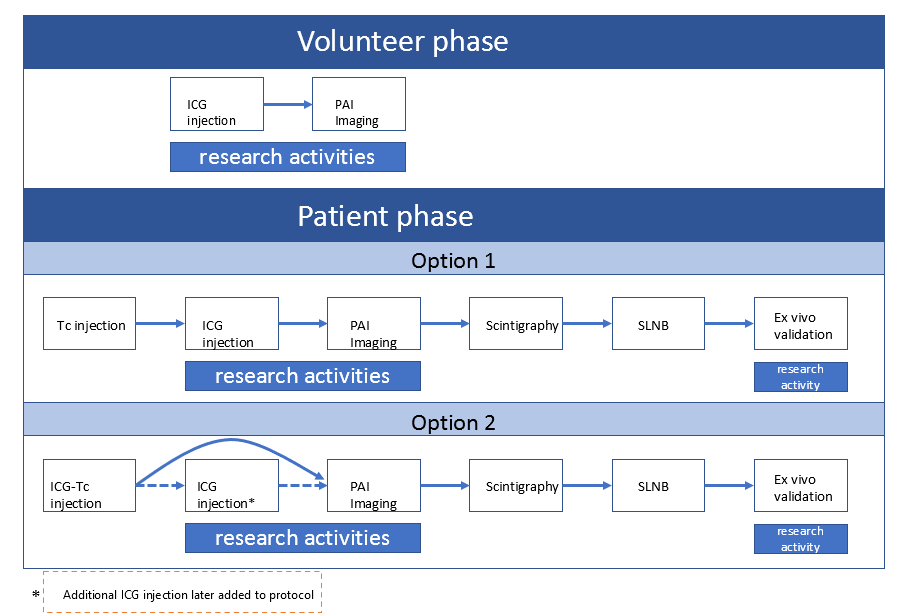


Supplementary Figure 1: Flowchart of the volunteer and patient phases.


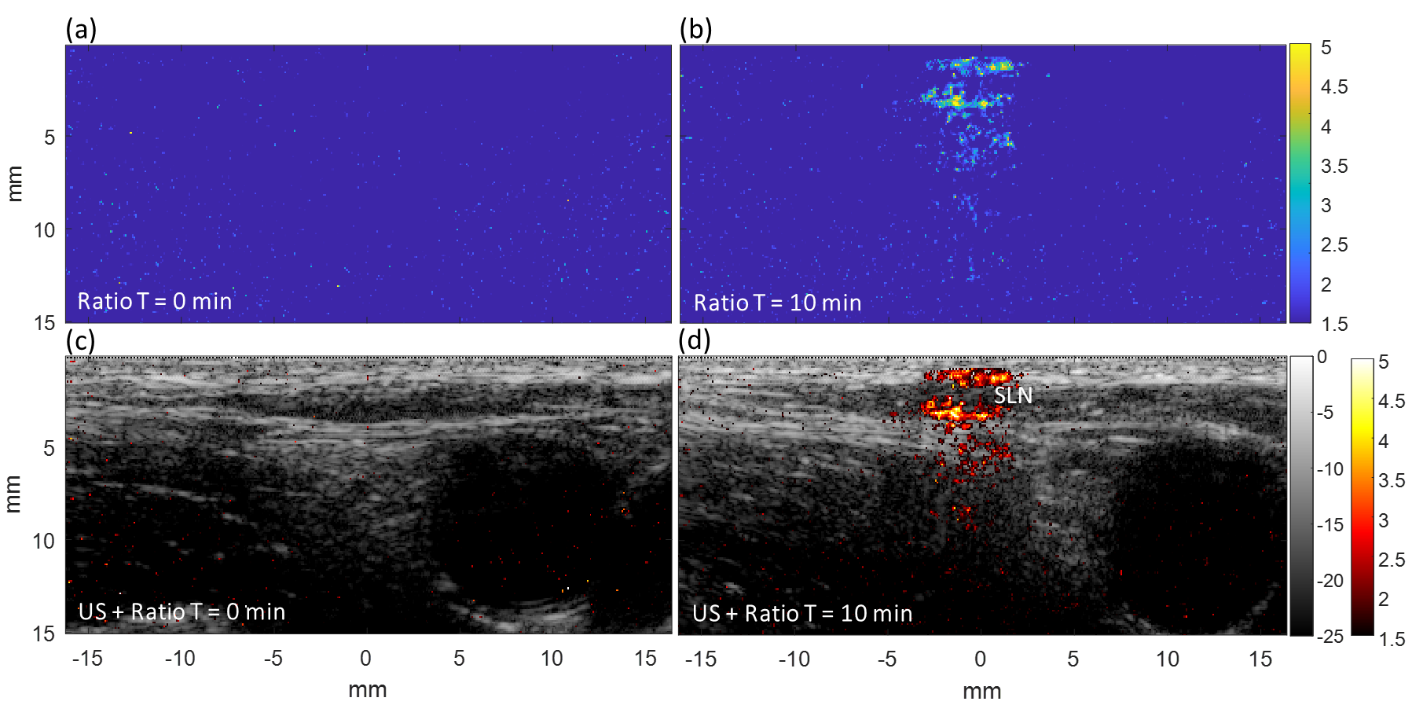


Supplementary Figure 2: Photoacoustic ratio images of an inguinal sentinel lymph node (SLN) in a healthy volunteer before (a) and 10 minutes after (b) indocyanine green (ICG) administration. The ICG rapidly accumulates in the SLNs, allowing for photoacoustic visualization of SLNs shortly after the administration. An overlay of the ratio image onto the corresponding ultrasound image is shown in (c) and (d).


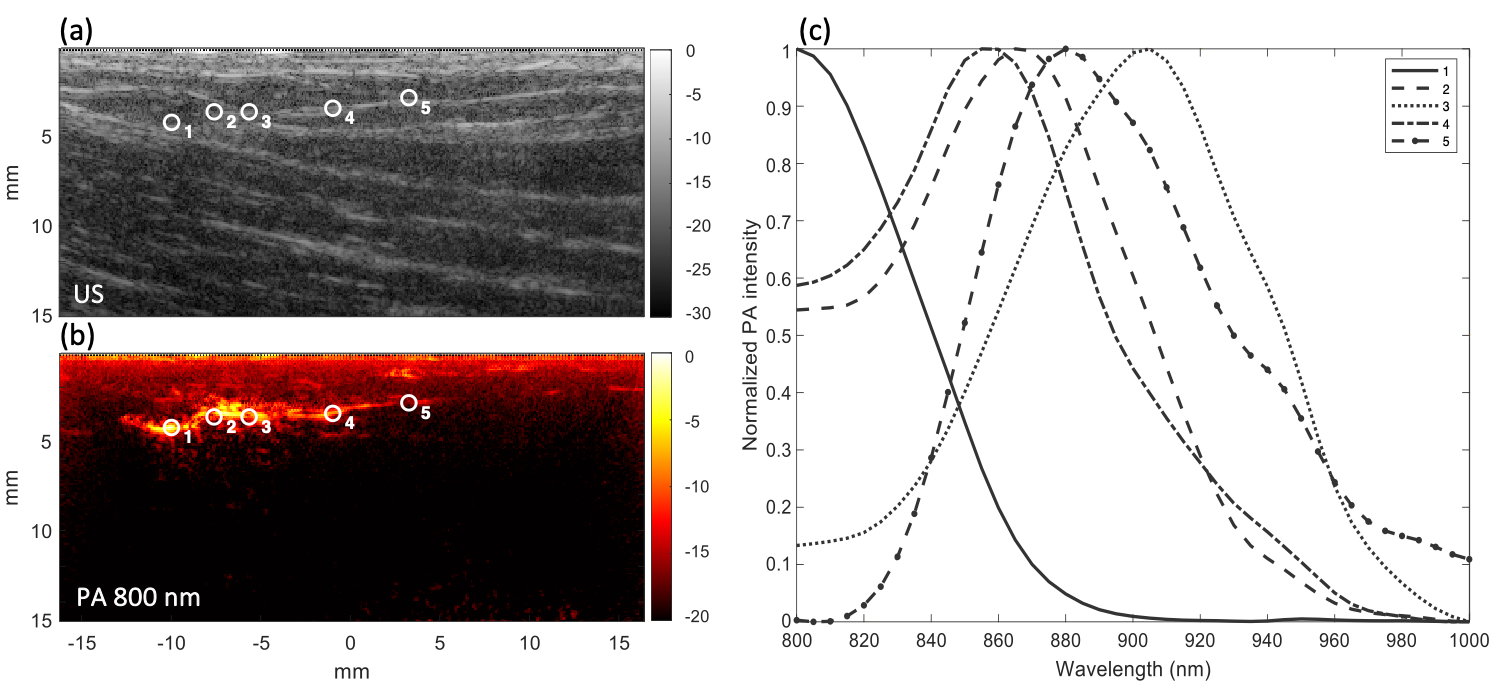


Supplementary Figure 3: Ultrasound (a) and photoacoustic image at 800 nm (b) at the injection site, minutes after indocyanine green (ICG) injection. Various regions are marked, and the average photoacoustic spectra (min-max scaled) are visualized in (c), showing variation in the ICG absorption peak.
